# Process Characterization and Determinants of CD3⁺ Cell Yield During Autologous Leukapheresis for CAR-T Cell Manufacturing: A Single-Centre Experience from Western India

**DOI:** 10.1016/j.htct.2026.108545

**Published:** 2026-10-19

**Authors:** Priti Desai, Anisha Navkudkar, Aswathi Suresh, Manju Sengar, Hasmukh Jain, Bhausaheb Bagal, Lingaraj Nayak, Alok Shetty, Rahul Purwar, Atharva Karulkar, Devanshi Kalra, Smriti Ravikumar, Prashant Tembhare

**Affiliations:** a Department of Transfusion Medicine, Tata Memorial Hospital, Mumbai, India; b Homi Bhabha National Institute, Training School Complex, Anushakti Nagar, Mumbai 400094, India; c Department of Medical Oncology, Tata Memorial Hospital, Mumbai, India; d Indian Institute of Technology, Bombay, India; e Immunoadoptive Cell Therapy Private Limited (ImmunoACT), India; f Advanced Centre for Treatment, Research and Education in Cancer, Tata Memorial Center, Mumbai, India

**Keywords:** Car t-cell therapy, Hematological parameters, Blood component transfusions

## Abstract

**Background:**

Chimeric Antigen Receptor T-cell **(**CAR T-cell) therapy is a promising treatment option for patients with refractory or relapsed B-cell malignancies. The success of this therapy depends on the efficient collection of lymphocytes (CD3^+^ T cells) through apheresis. This study analyzed the factors influencing CD3^+^ cell counts in apheresis products for CAR T-cell therapy.

**Methods:**

This exploratory analysis evaluated patients enrolled in the registration phase I/II trials of talicabtagene autoleucel at a tertiary care oncology centre in Western India. Patients were enrolled after obtaining informed consent. The study was approved by the Institutional Ethics Committee and the Central Drugs Standard Control Organisation (CDSCO), and registered with the Clinical Trials Registry of India. A total of 48 autologous lymphocytapheresis procedures were performed on 44 patients with refractory or relapsed lymphoma (predominantly diffuse large B-cell lymphoma) or B-acute lymphoblastic leukemia. Pre-apheresis hematological parameters and flow cytometry were used to estimate absolute lymphocyte counts and peripheral blood CD3^+^ cell counts. Predictive factors for CD3^+^ cells in the product were analyzed using the Pearson correlation coefficient and regression models by the Statistical Package for Social Sciences software (Version 25.0, Armonk, NY: IBM Corp.), and p-values <0.05 were considered statistically significant.

**Results:**

Pre-apheresis parameters like total leukocyte count, absolute lymphocyte count, and circulating CD3⁺ cell count were all positively correlated with the absolute CD3^+^ cell yield in the apheresis product (p < 0.05). Importantly, these associations remained significant in multivariate analysis, highlighting their independent predictive value for the harvested CD3^+^ count. Additionally, lower pre-apheresis hematocrit and higher platelet levels were independently associated with reduced CD3^+^ recovery. Notably, the targeted CD3^+^ cell dose was successfully achieved in 96 % (46/48) of collections.

**Conclusion:**

Pre-apheresis total leukocyte count, absolute lymphocyte counts, CD3^+^ cells, hematocrit, and platelet counts are key factors influencing the successful collection of CD3^+^ cells during apheresis. Understanding these variables is crucial for optimizing lymphocyte collection for CAR T-cell manufacturing.

## Introduction

Chimeric antigen receptor T-cell (CAR T-cell) therapy is an innovative immunotherapy that has demonstrated significant efficacy in treating refractory or relapsed (r/r) hematologic malignancies [1,2].

CAR T-cell manufacturing involves harvesting non-stimulated peripheral blood mononuclear cells (MNCs) via leukapheresis, followed by T-cell selection, activation, viral vector-mediated CAR gene transduction, and *ex vivo* expansion of the final cellular product [3,4]. One of the critical steps in CAR T-cell therapy is the collection of sufficient lymphocytes, specifically CD3^+^ T cells, through apheresis which aims at maximizing the collection of CD3^+^ T cells [3]. The quality of the apheresis product collected determines the success of CAR T-cell production and also has a huge impact on clinical outcomes [5]. Studies have shown that a minimum of 6 × 10^8^ CD3^+^ cells is required for successful manufacturing of the target CAR T-cell therapy dose [6,7]. Wada et al. demonstrated that CD3^+^ cell counts at leukapheresis can be a surrogate marker for the quality of T cells, efficacy of CAR T-cell production, and *in vivo* environment [8]. Although collection of a sufficient number of CD3^+^ cells can be attained by optimising procedure parameters during leukapheresis even in patients with low CD3^+^ cell counts, the subsequent *ex vivo* manufacturing process of CAR T cells, where T cells are activated, transduced with CAR, and then expanded, can be difficult in patients with low CD3^+^ cell counts at the time of leukapheresis [8].

The hematological parameters considered for hematopoietic progenitor cell collection may not apply to MNC collections because non-mobilized patients often have low white blood cell (WBC) counts, making isolation of target lymphocytes challenging [7]. Furthermore, compared with healthy donors, patients referred for CAR T-cell therapy are often heavily pretreated and frequently exhibit lymphopenia, elevated levels of circulating malignant cells, or both [7]. It has been observed that the target cells required here are mature lymphocytes, which are smaller and denser, potentially making apheresis more of a technical challenge because of the difficulty in establishing the red blood cell-WBC interface in the centrifuge [9]. All these factors may influence the collection of sufficient lymphocytes and form a major barrier to the overall success of CAR T-cell therapy. Though it has been demonstrated that the collection of the MNC layer is a safe and efficient method for collecting T cells necessary to produce CAR T-cell products, the optimal apheresis collection parameters have not been defined [9]. Factors such as pre-apheresis hematological parameters including lymphocyte counts and procedural variables can significantly influence the yield of CD3^+^ cells. The recommended pre-apheresis thresholds for various hematological parameters are hemoglobin >8 g/dL, hematocrit >0.24, absolute lymphocyte count (ALC) >0.2 × 10^9^ /L and platelet count >30 × 10^9^/L [10].

The present study discusses experience from a tertiary care oncology centre from western India regarding the factors affecting CD3^+^ cell counts in the apheresis product for autologous CAR T-cell therapy.

## Material and methods

### Study design

This prospective observational study was conducted between January and December 2023 at a tertiary care oncology hospital in Western India. The study was approved by the Institutional Ethics Committee (IEC) and registered with Clinical Trial Registry of India (CTRI: CTRI/2021/04/032727 and CTRI/2022/12/048211). The study population comprised adolescent and adult patients (aged >15 years) diagnosed with r/r B-cell non-Hodgkin lymphoma or B-acute lymphoblastic leukemia. The patients planned for lymphocyte harvest by apheresis were referred to the Department of Transfusion Medicine for venous assessment (peripheral/central) a few days prior to the date of apheresis. The procedure was performed after obtaining written informed consent from the patient or guardian in the case of minors. The primary objective of this study was to analyze the correlation between pre-apheresis hematological parameters, such as total leukocyte count (TLC), ALC, and CD3^+^ cell count, with the total CD3^+^ cell yield of the apheresis product. The impact of pre-apheresis hematocrit levels and platelet counts on the efficiency of CD3^+^ cell collection (and their role in achieving the target CD3^+^ cell count) was also analysed.

### Parameters analysed

#### Hematological parameters of the patient

All patients underwent a thorough hematological evaluation a day prior to or on the same day of apheresis. This included a complete blood count (CBC) to determine hemoglobin levels, hematocrit, platelet count, TLC, lymphocyte percentage and ALC. Additionally, Flow cytometry was performed to estimate the absolute count of CD3^+^ cells in the peripheral blood.

#### Procedure parameters

All apheresis procedures were conducted using the Spectra Optia Apheresis System (Terumo BCT, Japan) with the continuous mononuclear cell collection (cMNC) protocol. The procedures aimed to collect a target yield of 6 × 10^8^ CD3^+^ cells per unit. The procedures were performed using acid citrate dextrose (ACD-A) solution as an anticoagulant. The volume of blood processed during each procedure, the duration of the procedure, and the volume of ACD used were recorded and evaluated.

#### Product parameter

The apheresis product was analyzed for its volume, TLC, ALC, and CD3^+^ cell count. CD3^+^ cells in the product were quantified using flow cytometry. The collection efficiency for CD3^+^ cells was calculated as the percentage of CD3^+^ cells in the apheresis product relative to the total CD3^+^ cells available in the peripheral blood before the procedure.

The formula used to calculate collection efficiency is [11]:

$$\text{Collection}\;\text{efficency}\;2\left(\%\right)=\frac{\text{Product}\;\text{CD}3^{+}\;\text{concentration}\times \text{Product}\;\text{volume}\times 100}{\text{Peripheral}\;\text{blood}\;\text{CD}3^{+}\;\text{concentration}\times \text{Processed}\;\text{blood}\;\text{volume}}$$

Where:

• Product CD3⁺ concentration = CD3⁺ cells/µL in the apheresis product
• Product volume = final collection volume (mL)
• Peripheral blood CD3⁺ concentration = pre-apheresis CD3⁺ cells/µL
• Processed blood volume = total whole blood processed during leukapheresis, excluding anticoagulant volume (mL)

#### Intra-apheresis absolute lymphocyte count to predict final collection volume required to achieve a target yield of 6 × 10⁸ CD3⁺ cells per apheresis product

The patient's baseline peripheral blood CD3⁺ percentage, determined by flow cytometry prior to leukapheresis, was utilized to develop a practical intra-procedural approach for estimating the collection volume required to achieve the target CD3⁺ cell yield of 6 × 10⁸ cells per apheresis product. During the procedure, an intra-apheresis sample was obtained from the product line and analyzed for ALC. Assuming that the proportion of CD3⁺ cells within the lymphocyte population remained comparable to the baseline peripheral blood CD3⁺ percentage, the CD3⁺ cell concentration in the apheresis product was estimated using the following relationship:

$$\text{Estimated}\;\text{CD}3^{+}\;\text{cells}/\text{mL}=\text{Intra}-\text{apheresis}\;\text{ALC}\times \text{Baseline}\;\text{CD}3^{+}\;\text{percentage}$$

The estimated CD3⁺ cell concentration was then used to calculate the projected CD3⁺ cell yield at any given collection volume:

$$\text{Predicted}\;\text{CD}3^{+}\;\text{cell}\;\text{yield}=\text{Estimated}\;\text{CD}3^{+}\;\text{cells}/\text{mL}\times \text{Product}\;\text{volume}\;\text{collected}$$

Based on the estimated CD3⁺ cell concentration, the total collection volume required to achieve the target yield of 6 × 10⁸ CD3⁺ cells was calculated as:

$$\text{Required}\;\text{collection}\;\text{volume}\;(\text{mL})=\text{Target}\;\text{CD}3^{+}\;\text{cell}\;\text{dose}\;÷\;\text{Estimated}\;\text{CD}3^{+}\;\text{cells}/\text{mL}$$

This real-time estimation enabled assessment of whether the target CD3⁺ cell dose was likely to be achieved and facilitated adjustment of collection duration or processed blood volume during the procedure when necessary. Predicted CD3⁺ cell yields and calculated collection volumes were subsequently compared with the actual yields in the apheresis product and collection volumes to evaluate the feasibility of this approach.

### Statistical analysis

The data were analyzed using Statistical Package for Social Sciences software (SPSS Version 25.0, Armonk, NY: IBM Corp.). Descriptive statistics were used to summarize patient demographics, pre-apheresis hematological parameters, and apheresis product characteristics. The Pearson correlation coefficient was used to assess the relationship between pre-apheresis parameters and the yield of CD3^+^ cells in the apheresis product. Multivariable linear regression analysis was conducted to explore the independent association of pre-apheresis variables with CD3⁺ cell yield and to identify potential predictors of collection outcomes. A p-value <0.05 was considered statistically significant.

## Results

A total of 48 autologous lymphocytapheresis procedures were performed on 44 patients of whom 86% (n = 38) were male and 14% (n = 6) were female (Table 1). The median age and body weight of the patients were 43 years (range: 16–72 years) and 66 kg (range: 43–91.8 kg), respectively. Most of the patients were diagnosed with diffuse large B-cell lymphoma (DLBCL) (n = 24/44; 54.5%) and B-cell acute lymphoblastic leukemia (n = 13/44; 30%).

**Table 1:** Patient and Apheresis Product Characteristics.

Characteristic	Values
**Patient demographic details**	
Age (years) - Median (Range)	43 (16–72)
Body weight (kg) - Median (Range)	66 (43–91.8)
Total blood volume (mL) - Median (Range)	4540 (3010–6426)
Sex - n (%)	44
Male	38 (86)
Female	6 (14)
Diagnosis	
Lymphoma - n (%)	31 (70)
• DLBCL	24 (54.5)
• Others	7 (15.5)
Leukemia:	
• B-ALL	13 (30)
**Patient pre-apheresis hematological parameters**	
Hemoglobin (g/dL) - Median (IQR)	10.4 (9.5- 11.4)
Hematocrit (%) - Median (IQR)	34 (30.5- 36.8)
Platelet counts (× 10^9^/L) - Median (IQR)	162 (102- 251)
Patient pre-apheresis Flow cytometry analysis	
Total Leucocyte counts, TLC (/µL) - Median (IQR)	4337 (2832- 6498)
Absolute Lymphocytes (/µL) - Median (IQR)	1002 (849- 1496)
CD3+ cells (/µL) - Median (IQR)	808 (670- 1120)
**Apheresis sessions**	
One	40
Two	4
**Lymphocytapheresis procedures parameters**	
Venous access	
Central	29
Peripheral	19
Collection mode - cMNC procedures	48
Total blood volume processed (mL) - Median (IQR)	6188 (5101–7574)
Duration (minutes) - Median (IQR)	170 (138–215)
Volume of ACD used (mL) - Median (IQR)	565 (463–674)
Collection Efficiency for CD3+ cells in apheresis product (%) - Median (Range)	34 (11–105)
**Apheresis product parameters**	
Product volume (mL), Median (IQR)	130 (100–172)
Total Leucocyte counts, TLC (/µL) - Median (IQR)	41,135 (30,342- 56,740)
Absolute Lymphocytes, ALC (/µL) - Median (IQR)	21,780 (13,315- 29,995)
CD3+ cells (/µL), Median (IQR)	5545 (3872- 8075)
Post-manufacturing infusion product parameters	
CD3+ cell yield (× 10^8^/unit)	6.44 (3–16.7)
CD3+ cell dose (× 10^6^/kg)	10.67 (4–21)

DLBCL: Diffuse large B-cell lymphoma; B-ALL: B-cell acute lymphoblastic leukemia; IQR: Interquartile range; cMNC: Continuous Mononuclear Cell Collection.

### Pre-apheresis hematological parameters

The median hemoglobin level, hematocrit level, platelet count, TLC and ALC of the patient were 10.4 g/dL, 34%, 162 × 10^9^/L, 4.337 × 10^3^/µL and 1.002 × 10^3^/µL respectively while the median CD3^+^ cell count was 808/µL as summarised in Table 1.

### Apheresis procedure details

The procedure was conducted through central venous access in 29 procedures (65.9%) and by peripheral venous access in 19 procedures (43.1%). The median volume of blood processed and volume of ACD used during the apheresis procedure were 6188 mL (range: 5101–7574 mL) and 565 mL (range:463–674 mL), respectively. The median duration of the procedure was 170 min (range: 138–215 min). (Table 2, Table 3)

**Table 2:** Comparison between procedural parameters in Lymphoma and Leukemia patients.

Procedure	Lymphoma (n = 31 patients, 35 procedures)	Leukemia (n = 13 patients, 13 procedures)
**Patient pre-apheresis hematological parameters**		
Hemoglobin (g/dL) - Median (IQR)	10.5 (9.5–11.6)	10.3 (9.6–11.1)
Hematocrit (%) - Median (IQR)	35.15 (30.8–37.1)	32.7 (30.67–35.35)
Platelet counts (× 10^9^/L) - Median (IQR)	183.5 (105.25–257.75)	157 (69.75–169.25)
**Patient pre-apheresis flow cytometry analysis**		
Total Leucocyte counts, TLC (/µL) - Median (IQR)	4801.5 (3063.5–6403.5)	3149.5 (2425.25–6651.5)
Absolute Lymphocytes (/µL) - Median (IQR)	1060 (886.25–1500)	971 (713.75–1098.25)
CD3+ cells (/µL) - Median (IQR)	464 (677.5–1141.5)	779 (659.25–1006.75)
**Apheresis sessions**		
One	27	13
Two	4	
**Procedural parameters**		
Total blood volume processed (mL) - Median (IQR)	6200 (5476–7435.25)	5370 (4148–7681)
Duration (minutes) - Median (IQR)	170.5 (144.5–219.25)	156 (111–186)
Volume of ACD used (mL) - Median (IQR)	576.5 (496–669)	488.5 (402.75–698.25)

**Table 3:** Pre-apheresis decision toolkit.

Parameter	Favorable observation in successful collections	Practical implication
TLC	Higher baseline TLC	- Consider timing the lymphocyte collection after count recovery
		- Consider delaying collection if severe lymphopenia
ALC	Higher counts	Better CD3+ yield expected
Peripheral CD3+	Higher counts	Lower TBV may suffice
Hematocrit	Avoid severe anemia (Maintain Hb >7 *g*/dL)	Better interface stability
Platelets	Avoid excessively high counts	May affect interface, Monitor collection quality

TLC: Total leukocyte count; ALC: Absolute lymphocyte count; TBV: Total blood volumes.

Of the 44 patients included in the study, four patients required a second leukapheresis procedure to achieve the target CD3⁺ cell dose for CAR T-cell manufacturing. Compared with patients who achieved the target in a single procedure, these patients demonstrated relatively lower pre-apheresis ALC and circulating CD3⁺ cell counts. These findings suggest that patients with lower baseline ALC and CD3⁺ counts may be at increased risk of suboptimal collection and could potentially benefit from individualized collection planning strategies.

Lymphocyte collection efficiency averaged 34% (range: 11–105%). Collection efficiency values exceeding 100% may indicate lymphocyte redistribution during prolonged leukapheresis, as cells enter circulation from the spleen, lymphoid tissues, or marginal pools. Consequently, the collected CD3⁺ cells may appear greater than the number estimated from the baseline sample, resulting in collection efficiency values >100%.

### Product characteristics

The analysis of the apheresis product revealed that the median product volume was 130 mL (range: 100–172 mL). The median TLC in the apheresis product was 41.1 × 10⁹ cells/L (range: 30.3–56.7 × 10⁹ cells/L) while the ALC was 21.8 × 10⁹ cells/L (range: 13.3–30.0 × 10⁹ cells/L) and the CD3^+^ cell count was 5.55 × 10⁹ cells/L (range: 3.87–8.08 × 10⁹ cells/L)

### Post- manufacturing infusion

The post-manufacturing infusion product contained a median CD3^+^ cell yield of 6.44 × 10^8^/unit (range: 3–16.7 × 10^8^/unit) and the CD3^+^ cell dose administered to the patients was 10.67 × 10^6^/kg (range: 4–21 × 10^6^/kg).

This study found a significant positive correlation between pre-apheresis TLC, ALC, and CD3^+^ cell counts with the absolute number of CD3^+^ cells in the apheresis product (r = 0.299; p = 0.039, r = 0.483; p = 0.001, and r = 0.434; p = 0.002, respectively) (Figure 1). This indicates that higher pre-apheresis lymphocyte and CD3^+^ cell counts are associated with a higher yield of CD3^+^ cells in the apheresis product. Multivariable regression analysis demonstrated non-statistically significant trends towards lower pre-apheresis hematocrit levels and higher platelet counts being associated with reduced apheresis product CD3^+^ cell yield. Regression analysis was used to identify independent predictors. The target CD3^+^ cell count of 6 × 10^8^/unit was achieved in 96% (46/48) of the apheresis procedures. This high success rate indicates the efficacy of the apheresis process in collecting sufficient CD3^+^ cells for CAR T-cell manufacturing.

**Figure 1:**
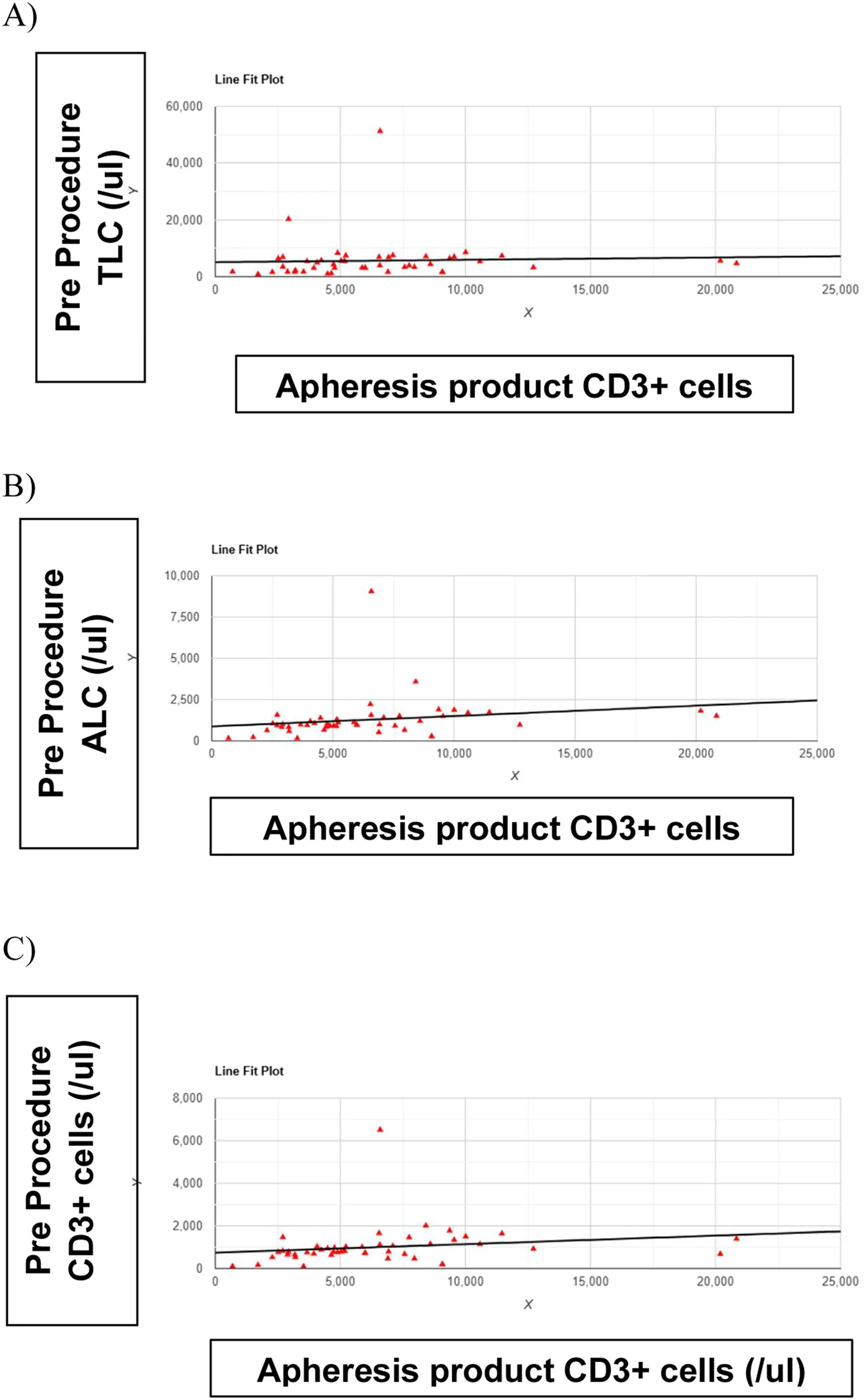
Total leukocyte count (TLC), absolute lymphocyte count (ALC), and pre-procedure peripheral blood CD3⁺ count were all positively correlated with apheresis CD3+ product yield (r = 0.299; p = 0.039, r = 0.483; p = 0.001, r = 0.434; p = 0.002, respectively).

## Discussion

This study primarily characterizes the leukapheresis process and identifies key predictors of CD3⁺ cell yield in adult patients with B-cell malignancies undergoing CAR T-cell manufacturing [[11], [12], [13]]. Notably, most procedures required processing of only about 1.4 of the total blood volumes (TBV) to meet the target of 6 × 10⁸ CD3⁺ cells, substantially less than the 2–4 TBV typically reported elsewhere. The study identified several key pre-apheresis hematologic parameters that strongly predicted CD3⁺ cell yield: the TLC, ALC, and peripheral blood CD3⁺ count were all highly correlated with final product yield. Importantly, these associations remained significant in multivariate models. Additionally, two independent predictors emerged i.e. low hematocrit (<26%) and high platelet count (>30 × 10^9^/L), both of which were associated with reduced CD3⁺ recovery. A low hematocrit concentration likely impairs cell separation efficiency, while elevated platelets may lead to clotting or disrupt interface clarity during centrifugation. Anticoagulation was managed with citrate dextrose solution A (ACD-A), with a typical blood-to-ACD ratio of 12:1, maintaining circuit patency throughout [14]. Lymphocyte collection efficiency averaged 34% (range: 11–105%), compared to 43% (range: 0.3–252%) in previous reports. Efficiency was calculated over the first 4–5 procedures, then used to guide TBV targets for subsequent collections. The procedures exhibited an excellent safety profile, with no adverse events like bleeding, hypotension, or allergic reactions. A total of 96% of the procedures reached the target CD3⁺ count demonstrating the importance of pre-apheresis evaluation to ensure adequate yields. Despite these promising outcomes, leukapheresis yields a mixed MNC population, including monocytes that can differentiate into macrophages and potentially phagocytose CAR T beads, reducing the viable T cell yield [15,16]. This challenge is well-documented in hematopoietic stem cell collections and remains a concern here. To address monocyte contamination, we propose the use of mid-procedure complete blood counts (CBCs) as a practical checkpoint. Real-time CBC data could inform dynamic adjustments to collection parameters such as modifying processed TBV or adjusting the parameters to limit monocyte content and improve product quality.

Pre-apheresis optimization strategies may improve successful lymphocyte collections. Whenever clinically feasible, leukapheresis may be scheduled after hematological recovery from recent chemotherapy, while avoiding profound lymphopenia. Adequate hematocrit levels may improve interface stability during centrifugation, and patients with low circulating CD3⁺ counts may benefit from higher planned processed TBV.

## Conclusion

Pre-apheresis hematologic parameters, including TLC, ALC, peripheral blood CD3⁺ cell count, hematocrit, and platelet count, are important determinants of successful CD3⁺ cell harvest during leukapheresis for CAR T-cell manufacturing. Among these, ALC and circulating CD3⁺ cell counts demonstrated significant predictive value for CD3⁺ yield in the apheresis product, supporting their utility in pre-procedure assessment and collection planning. The predictive framework developed in this study provides a practical approach for identifying patients at risk of suboptimal collection and may assist in tailoring procedural strategies such as timing of leukapheresis and blood volume processing. A clear understanding of these variables can help optimize lymphocyte collection, improve manufacturing success, and enhance the overall delivery of CAR T-cell therapy for patients with relapsed or refractory hematologic malignancies. This study represents one of the earliest reports from India to characterize leukapheresis processes and develop predictive approaches for CAR T-cell collection.

## Data availability

The data that support the findings of this study are available from the corresponding author upon reasonable request

## Conflicts of interest

Immunoadoptive Cell Therapy Private Limited (ImmunoACT).
